# Enhancing screening, brief intervention, and referral to treatment among socioeconomically disadvantaged patients: study protocol for a knowledge exchange intervention involving patients and physicians

**DOI:** 10.1186/1472-6963-13-108

**Published:** 2013-03-22

**Authors:** Ginetta Salvalaggio, Kathryn Dong, Christine Vandenberghe, Scott Kirkland, Kelsey Mramor, Taryn Brown, Marliss Taylor, Robert McKim, Greta G Cummings, T Cameron Wild

**Affiliations:** 1 Department of Emergency Medicine, University of Alberta, Room 565 CSC, Royal Alexandra Hospital, 10240 Kingsway Avenue, Edmonton, AB T5H 3V9, Canada; 2 Department of Emergency Medicine, University of Alberta, Edmonton, AB, Canada; 3 University of Toronto, Toronto, Ontario, Canada; 4 Streetworks, 10116-105 Ave, Edmonton, AB, T0B 4J0, Canada; 5 Edmonton South Side and Edmonton West Primary Care Networks, Edmonton, AB, Canada; 6 Faculty of Nursing, 5–110 ECHA, University of Alberta, Edmonton, AB, T6G 0C1, Canada; 7 School of Public Health, 3–277 ECHA, University of Alberta, Edmonton, AB, T6G 2G4, Canada; 8 Department of Family Medicine, University of Alberta, 1702 College Plaza, Edmonton, AB, T6G 2C8, Canada

**Keywords:** Addiction, Screening, Brief intervention, Family medicine, Emergency medicine, Underserved patients, Patient engagement

## Abstract

**Background:**

Screening, Brief Intervention, and Referral for Treatment (SBIRT) is an effective approach for managing alcohol and other drug misuse in primary care; however, uptake into routine care has been limited. Uptake of SBIRT by healthcare providers may be particularly problematic for disadvantaged populations exhibiting alcohol and other drug problems, and requires creative approaches to enhance patient engagement. This knowledge translation project developed and evaluated a group of patient and health care provider resources designed to enhance the capacity of health care providers to use SBIRT and improve patient engagement with health care.

**Methods/Design:**

A nonrandomized, two-group, pre-post, quasi-experimental intervention design was used, with baseline, 6-, and 12-month follow-ups. Low income patients using alcohol and other drugs and who sought care in family medicine and emergency medicine settings in Edmonton, Canada, along with physicians providing care in these settings, were recruited. Patients and physicians were allocated to the intervention or control condition by geographic location of care. Intervention patients received a health care navigation booklet developed by inner city community members and also had access to an experienced community member for consultation on health service navigation. Intervention physicians had access to online educational modules, accompanying presentations, point of care resources, addiction medicine champions, and orientations to the inner city. Resource development was informed by a literature review, needs assessment, and iterative consultation with an advisory board and other content experts. Participants completed baseline and follow-up questionnaires (6 months for patients, 6 and 12 months for physicians) and administrative health service data were also retrieved for consenting patients. Control participants were provided access to all resources after follow-up data collection was completed. The primary outcome measure was patient satisfaction with care; secondary outcome measures included alcohol and drug use, health care and addiction treatment use, uptake of SBIRT strategies, and physician attitudes about addiction.

**Discussion:**

Effective knowledge translation requires careful consideration of the intended knowledge recipient’s context and needs. Knowledge translation in disadvantaged settings may be optimized by using a community-based participatory approach to resource development that takes into account relevant patient engagement issues.

**Trial registration:**

Northern Alberta Clinical Trials and Research Centre #30094

## Background

Misuse of alcohol and other drugs has far-reaching impacts on both public and individual health [1,2]. Approximately fifteen percent of adults are problem drinkers and twelve percent of adults use illicit drugs; a significant proportion of adults also experience harm due to alcohol (seven percent) or drugs (three percent) [1,2]. Addiction is associated with higher premature and overall mortality [3]. Morbidity arises from several conditions ranging from infectious, cardiorespiratory, and gastrointestinal diseases to trauma and comorbid mental illness and chronic pain [4,5]. Despite this increased burden of illness, people who live with addiction are less likely to receive preventive health care and are more likely to present with more acute and severe illness than the general population [6,7]. Increased health care costs are not the only financial impact; social and law enforcement costs are also considerable [8].

Primary care is uniquely placed to intervene in alcohol and other drug misuse through prevention, case finding, preliminary management, referral, and long term follow-up efforts. Emergency medicine settings can also play an important role in assessment, especially for patients who do not have access to a primary care provider or who present with acute substance-related injuries. Screening, Brief Intervention, and Referral for Treatment (SBIRT) has been proposed as a structured approach to the assessment of addictive behaviours in these service contexts [9]. This three-step strategy can be as brief as eight to ten minutes and can involve one or more health care team members; it incorporates screening for substance misuse using validated instruments, provision of brief intervention for positive screens using principles of motivational interviewing [10], and referral for specialized addiction treatment for those individuals who meet clinical criteria for substance dependence. A large body of efficacy and effectiveness studies support the use of SBIRT in primary care and emergency medicine settings to decrease overall alcohol consumption and increase addiction treatment uptake and retention [11,12]. The evidence for SBIRT’s impact on consumption, access to specialized treatment, and morbidity for drugs other than alcohol in the general population is more limited but may be appropriate in selected populations.

Despite the documented benefits of SBIRT and endorsements by prominent public health, primary care, emergency medicine and addiction medicine organizations [13-17], implementation in primary care and emergency care settings has been limited [18-20]. SBIRT is often viewed as time- and resource-intensive, especially in traditional care settings with service-based remuneration and limited access to multidisciplinary team members [21]. Other preventive strategies (e.g. cancer screening, blood pressure monitoring) and the management of acute issues may hold higher priority [22]. Health care providers also cite limited training in this area and lack of confidence in their abilities [23]. Further, some settings have minimally integrated addiction services with other health care services, thus limiting the follow-up support available to primary and emergency care teams. Health service providers and researchers have acknowledged these barriers, and there is increasing interest in developing and testing interventions designed to enhance uptake of SBIRT into routine care. Some of these knowledge translation (KT) initiatives involve examining the impact of curriculum changes on practitioners’ SBIRT activities in the emergency department setting [24,25] and in community health clinics [26] as well as rural areas [27].

Barriers to SBIRT uptake are compounded by the obstacles vulnerable and underserved populations face when seeking and receiving care, or attempting to make positive behavioural changes. Factors such as housing, food security, employment, transportation, child care, health benefits, and literacy influence health and health care behaviour and are poorly addressed in traditional health care settings [28-30]. Once a patient successfully connects with health care services, the outcome of the health care encounter itself is shaped by several interpersonal considerations such as past care experiences, provider attitudes, the management of cultural differences and power imbalances, and potential discrimination [31,32]. Engaging patients in their health care improves health care quality and patient satisfaction [33]. Where social and health inequity exists, patient engagement is equally if not more important, and needs to consider the unique circumstances faced by this population. Positive strategies to engage vulnerable patients have included culturally appropriate models of care, case management, health navigation, community outreach, and advocacy group initiatives [34,35].

### Objectives

Unfortunately, extant research has not addressed these issues and barriers associated with effectively linking marginalized, disadvantaged populations to SBIRT interventions. Thus, our overall objectives were to develop a suite of KT resources designed to enhance uptake of SBIRT for alcohol and other drug misuse in this target population, and to examine the effectiveness of implementing these resources among patients and physicians. Specifically, the study was designed to detect meaningful changes in patient satisfaction as an interim outcome influencing eventual behavioural change; secondary outcomes included changes in substance use behaviour, treatment-seeking, health care use, physician use of and comfort with SBIRT, and physician attitudes about addiction.

## Methods/Design

### Overview and approach

**Figure 1:**
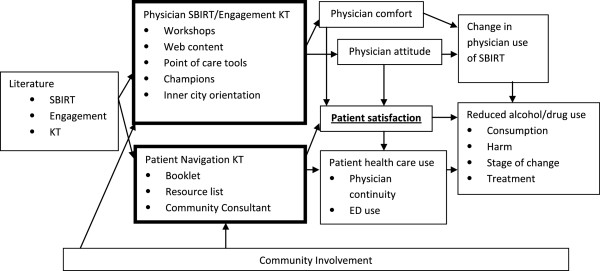
Project conceptual map.

This project took place in Edmonton, Canada, a city with a population of 817,498 persons [36]. Edmonton has a central downtown area where a disproportionate burden of the consequences of poverty and addiction reside. Several outreach organizations including the local harm reduction program, Streetworks, are located in this area. The project adopted a Community Based Participatory Research (CBPR) approach, wherein community stakeholders were involved from the inception of the research through to the project dissemination phase [37,38]. As such, the project was overseen by an advisory board consisting of researchers, KT experts, health care professionals, policy representatives, inner city community-based agencies, and community members. Community members co-developed the patient and physician interventions; provided input on participant recruitment, data collection, intervention delivery, and follow-up strategies; have agreed to assist with interpretation of findings and share findings with the community; and gave final approval for all project activities (Figure 1). Approval for the project was also obtained from the University of Alberta Health Research Ethics Board.

### Study design, sites, and statistical power

A nonrandomized, two-group, pretest-posttest, quasi-experimental intervention design with baseline, 6, and 12-month follow-up was used to evaluate the effectiveness of these resources. This design was chosen because the interactive nature of KT interventions limits the ability to apply randomized controlled trial principles such as blinding and allocation concealment. Randomizing the intervention by site was also not possible, as we chose to focus our efforts in specific geographic areas where our target patients were most likely to access care. Consequently, participants were allocated to either the intervention or control condition on the basis of the geographic location where they received health care. Intervention participants were recruited from two EDs and participating Family Medicine clinics affiliated with two Primary Care Networks (PCN, a group of physicians and multidisciplinary team members with the goal of improving comprehensive primary care access and care coordination). Control participants were recruited from a separate ED and Family Medicine clinics affiliated with a separate PCN in a different geographic area of the city. Additional participants unaffiliated with a specific intervention location of care were also recruited from community agencies (e.g. drop-in centres) to the control patient group to ensure a sufficiently large control sample.

Assuming a conservative proportion (e.g. 50%) of alcohol or drug using patients were satisfied with their care, a clinically meaningful increase in the proportion of satisfied patients of 20%, and a study power=0.80, we calculated that the minimum patient sample required to demonstrate a change in patient satisfaction as a result of our KT activities was 93 in each of the intervention and control groups. When adjusted for a predicted 25% or greater loss to follow-up based on other studies with similar patients [11,39], and taking into account the potential for heterogeneity between patients recruited from PCNs and EDs, we aimed to recruit 150 patients from the intervention PCNs, 150 patients from the intervention EDs, and 150 control patients, for a total sample size of 450.

### Participants

Both patients and physicians at the aforementioned study sites were invited to participate, since KT resources were developed for both audiences. Additional health care providers working alongside physicians were not formally recruited to participate; however, physician participants were encouraged to share KT resources with their multidisciplinary teams, and non-physician team members at participating locations were invited by the project team to attend KT activities and access resources.

#### Patients

Potential patient participants included those presenting to participating EDs, PCN-affiliated Family Medicine clinics, and community agencies. All patient participants were eligible for the study if they were 18 years or older, able to speak and understand English, self-identified as living in a low-income situation (income less than the current Canadian Low Income Cut Off [40]), stated they regularly use alcohol and/or drugs (based on positiveAlcohol Use Disorders Identification Test – Consumption (AUDIT-C) [41] or Drug Use Disorders Identification Test (DUDIT) [42] first question screen) and were in stable medical condition. Patients were excluded if they were unable to provide informed consent (due to intoxication, withdrawal, psychiatric symptoms), if they had come to the ED for a pre-arranged direct consult, or if they were in police custody or incarcerated.

#### Physicians

All physician participants worked in one of the study sites, expressed interest in participating in the study, and provided informed consent; no exclusion criteria were applied. Physicians were allocated to either the intervention or control condition on the basis of the usual geographic location where they provided health care. Intervention physicians were recruited from the same two EDs and two PCNs from which the intervention patient participants were recruited, as well as the University of Alberta Emergency Medicine and Family Medicine residency programs. Control physicians were recruited from the same ED and PCN as the control patient participants, as well as the University of Alberta General Internal Medicine residency program. Physician and medical resident sample sizes (300 and 200 respectively) were limited by the total population in each group. Sample sizes given are the approximate total number of physicians practicing within participating EDs and PCNs and approximate total number of residents currently training at the time of the project.

### Recruitment

#### Patients

Recruitment posters advertising the study were placed in prominent areas of the ED, Family Medicine waiting rooms, and community locations. Additionally a brief presentation and one page summary of the research project were made available to ED and Family Medicine staff.

During pre-set nine-hour data collection shifts at the EDs, registration clerks asked each patient arriving to the ED if they were interested in participating in a research study and completed a registration form for interested individuals. A Community Consultant (CC) was also available for certain shifts to assist with recruitment in the intervention sites. For interested patients who were assigned to a patient care space prior to being approached by the research team, the attending physician or nurse served as an intermediary for the study and asked for their permission to be approached. A Research Assistant (RA) then screened interested patients for low-income status and regular use of alcohol or drugs. The RA then reviewed other eligibility criteria, reviewed the study’s information letter with the patient, and asked them to sign the consent form if they agreed to participate in the study. Patients were then asked if they consented to having the study team track their health care utilization and addiction treatment data for the six months pre-enrolment and six months post-enrolment, and were asked to provide written consent on a separate consent form if they agreed to this aspect of the study. Patients were given the option to opt out of this aspect of the project, while continuing their participation in the larger study. Enrolled participants were subsequently provided with a baseline survey to complete with or without the assistance of the RA and in a private location.

For Family Medicine clinics, a Project Coordinator (PC) contacted and met with the physician (and/or other key clinic staff where deemed appropriate) to briefly discuss the patient arm of the study and how best to distribute baseline study materials to those interested. The PC tailored patient recruitment efforts at each clinic. In addition to recruitment posters, study packages were available in each clinic; patients had the option to complete baseline materials in the waiting room of their clinic, to complete their materials at home and mail in the completed survey, or to call the PC who would complete enrolment and baseline data collection on the phone or in person. Upon receipt of mailed study materials from participants choosing this means of enrolment, the PC contacted the patient, answered any questions about the study, confirmed their eligibility, and confirmed that they consented to have their health care utilization and addiction treatment activities tracked. Recruitment at community agency locations was conducted by the RA, and assisted by the CC. Information on the study as well as dates and times of data collection days were posted at each agency. On the data collection day, interested participants would sign up to meet with study staff at a specified time. The RA then met with potential participants at the specified time, and screened each participant for eligibility. Eligible participants were then enrolled in the study as described above.

Patient participants were reimbursed the standard local rate for research participation, namely $10CDN for baseline participation and $10CDN for follow-up participation.

#### Physicians

Physician participants were recruited using announcements at business meetings, via newsletters, and on local health services listservs. Letters introducing the project and inviting participation were sent to PCN and ED members via fax and mail. Residents were invited to participate in person via announcements during academic learning sessions. Physicians and residents were enrolled in the study upon receipt of their mailed or electronically completed informed consent and baseline survey materials.

To thank physicians for their participation, continuing professional development accreditation for the project KT activities was obtained from the Royal College of Physicians and Surgeons of Canada and the College of Family Physicians of Canada. In addition, participants were included in a draw for nine $50CDN gift cards to a local bookstore.

### Intervention

#### Patients

Patient-centered KT activities were initiated six months prior to physician-centered KT activities to minimize co-intervention effects. CBPR projects typically begin as a response to the community’s self-identified needs, involve community members in project design, implementation, interpretation, and communication, are sustainable over the long term, and build community research capacity. In this study, a patient engagement literature review, experience with past local inner city patient engagement projects, and consultation with community agencies and advocacy groups helped to determine the ideal content and resources for a patient-centered KT strategy. A community-written health navigation booklet and the CC role were developed to communicate core messages about health navigation and engagement.

The health navigation booklet was created in collaboration with the Streetworks harm reduction program, an organization that has long supported peer outreach activities including community produced outreach and education materials. The primary task in developing the booklet was recruitment of community authors with street expertise and insight. Streetworks assisted in identifying potential volunteers and after an initial group meeting to discuss the project, a core group was established. A research student and nurse acted as group facilitators. The next few weeks that followed focused on identifying knowledge gaps within the community and relevant advice for community members. The community members spoke of their previous health care related experiences while facilitators listened and reflected back ideas that could potentially assist their peers in a similar situation. From the discussion, themes were extracted to focus the written material (e.g. where and when to access care, what to expect during a visit, how to negotiate care with a health care team) and guide chapter or heading topics. The final stages of the project focused on devising booklet vignettes and formatting. The booklet was written in street-appropriate language and included personal story excerpts and illustrations to demonstrate key concepts from the perspective of the community. Prior to dissemination, content was externally reviewed by inner city health care providers and legal experts in the field. The final colors, illustrations, content and language were approved by the community members. The booklet was supplemented with a pocket guide of local health and social services.

Written KT resources are more effective when combined with other strategies. A community consultant with lived experience in the inner city joined the project to advise the core project team and liaise with community stakeholders. The CC’s primary role was to provide health navigation guidance for patient participants. The CC role combined peer outreach with the “champion” or content expert role described in KT literature. Participants were introduced to the CC through his outreach activities as well as a booklet insert describing the CC’s role in the project and his contact information.

#### Physicians

The Knowledge-to-Action cycle [43] has been successfully adapted to other physician KT projects; effective ways to change physician behaviours and practices apply multiple brief learning formats and include interactive small group practice, case-based learning, point-of-care reminders, audit and feedback, incentivization, and opinion leaders or “champions” [44]. We incorporated many of these strategies for the physician intervention. First, the literature on SBIRT and patient engagement was systematically reviewed to determine the optimal application of SBIRT in practice. Next, physician participants voluntarily completed a needs assessment survey to determine key learning needs and preferred learning formats. The research team also communicated with study sites to ensure that KT approaches would be appropriate for implementation in each practice setting. A series of KT resources were subsequently developed and participants were provided with frequent electronic updates and instructions on how to access these resources by the PC.

Core SBIRT and patient engagement content were initially offered in one to three hour workshops depending on the preferences of the participating site’s physicians. The workshop was available in podcast and webinar forms for participants unable to attend in person. Workshops offered some didactic material followed by facilitated small group SBIRT practice and a discussion of special populations and potential implementation strategies. Community members with lived addiction experience were available at a number of the sessions both to answer questions and to role-play scenarios with the participants. A number of web-based resources augmented the content offered at the workshops. Three videos were created to demonstrate the core messages in practice—two videos of SBIRT in Family Medicine and Emergency Medicine settings, and one video on patient perspectives about health care. Two online family medicine learning modules were also created, elaborating on such topics as comorbid mental illness, chronic pain, pregnancy, and ethical and legal issues in addiction care. In addition, an environmental scan of electronically available addiction assessment and patient engagement resources was undertaken. Informative external resources, along with the workshop podcast and slides, videos, and learning modules, were uploaded to Know-Mo, an addiction KT web platform based at the University of Alberta [45].

Reminder pocket guides were created for point-of-care use. An Edmonton-area resource list (including available shelters, detoxification facilities, residential and outpatient treatment programs, twelve-step meetings, etc.), as well as implementation “tip sheets” for family and emergency practice (advice on documentation, involvement of a multidisciplinary team, etc.), were created and disseminated. These point-of-care tools were also available in electronic form on the KT web platform.

Physician and other health care professional champions were identified for each participating site to help disseminate information about the project itself and the core messages. Champions participated in workshops, made themselves available to participants for future guidance, and were invited to liaise with the project team as needed. The CC was also identified as a community-based champion; he led a physical orientation to the inner city and several agencies involved in addressing addiction issues, and was also actively involved in the workshops.

Both physician and patient participants assigned to the control condition received usual care and/or education, however were granted full access to all KT resources upon completion of the intervention phase of the study and prior to broader dissemination.

### Measures

#### Patients

A baseline patient survey (Table 1) collected information regarding the patient’s demographics; satisfaction with care (the primary outcome) and rapport with their doctor; and experiences with pain, mental health, alcohol and drug use and attempts and quitting or limiting their substance use. The baseline patient survey incorporated validated instruments including the Alcohol Use Disorders Identification Test (AUDIT) [46], DUDIT [42], the Stages of Change Readiness and Treatment Eagerness Scale (SOCRATES) [47], the short form of the Health Care Climate Questionnaire (HCCQ) [48], and patient satisfaction items from the Alberta Physician Achievement Review program’s patient questionnaire [49]. Patients were also asked to provide their contact information, including a backup contact (e.g. family member, social agency) so that they could be contacted for the follow-up interview. Upon receipt of the completed baseline survey, intervention participants were provided with a copy of the health navigation booklet, contact information for the CC, and a local services pocket guide [50].

**Table 1:** Self reported participant variables (Patients)

Demographics	• Age
	• Gender
	• Ethnicity
	• Education level
	• Marital status
	• Number of children/custody status
	• Legal status (parole, probation, pending charges, etc.)
	• Years lived in Edmonton
	• Living situation (house, shelter, street, etc.)
	• Housing stability
	• Income/assets
	• Employment status
	
Health status	• Presence of comorbid health conditions (HIV, diabetes, etc.)
	• Health status
	• Vaccination status
	• Presence of chronic pain/pain severity/pain control
	• Patient Health Questionnaire-2 (depression screen)
	• Generalized Anxiety Disorder-2 scale (anxiety screen)
	
Satisfaction / rapport	• Presence of regular physician
	• Number of visits with regular physician
	• Disclosure of substance use to regular physician (yes or no)
	• Health Care Climate Questionnaire (short form)
	• Alberta Physician Achievement Review questionnaire satisfaction items
	
Substance use behaviour	• Alcohol Use Disorders Identification Test
	• Drug Use Disorders Identification Test
	• Stages Of Change Readiness and Treatment Eagerness Scale
	• Types of substances used
	• Injection drug use status
	
Substance use treatment	• Previous 6 month discussion re: substances with health care provider
	• Previous 6 month detoxification facility attendance
	• Previous 6 month residential treatment facility attendance
	• Previous 6 month methadone maintenance treatment program attendance
	• Previous 6 month 12 step meeting attendance
	
Project feedback	• Booklet use/usefulness
	• Booklet insert use/usefulness
	• Community consultant use/usefulness

Participants were contacted six months after completion of the baseline survey for a follow-up data collection session. The six-month patient survey repeated baseline survey items and also included items assessing whether or not participants used each KT resource and to what extent each resource was helpful if used. Control participants were provided with a copy of the health navigation booklet at the time of the follow-up session.

Participants who consented to having their health care and addiction treatment utilization tracked were asked to provide their Alberta Health Care unique identifier and date of birth to identify their care encounters within two available third party datasets. Alberta Health and Wellness manages provincial health care utilization data and Alberta Health Services-Addictions and Mental Health manages provincial addiction treatment data; both agreed to release anonymized data on administrative health data outcomes of interest, by intervention status, from consenting participants for the six months prior to and the six months following project enrolment. Key outcomes of interest (Table 2) focused on the timing and continuity of care and attempts at addiction treatment (ED use, Emergency Medical Services activation, night presentation, proportion of visits with one physician provider, addiction treatment enrolment and completion).

**Table 2:** Administrative dataset variables (Patients)

Addiction treatment services	• Start/completion of detoxification program
	• Start/completion of residential treatment program
	• Start/discontinuation of opioid dependency program
	• Start/completion of outpatient addiction programming
	• Primary substance for which treatment was sought
	
Ambulatory health services	• Service date and time
	• Triage level
	• Disposition
	• Major Ambulatory Category code (high level diagnostic grouping)
	• Ambulance transport (yes or no)
	
Inpatient health services	• Service date and time
	• Admit category (elective, urgent)
	• Length of stay
	• Disposition
	• Resource Intensity Weight
	• Case Mix Group

#### Physicians

Physician and resident participants were asked to complete a baseline survey via mail, phone, or online depending on their preference. The baseline survey (Table 3) asked physicians to provide information on their demographics; medical school training; comfort level and experience in treating those with substance abuse issues and for screening for such problems, as well as their attitudes towards those who use substances and those from low socioeconomic settings. The survey incorporated validated attitudinal instruments including the Short Understanding of Substance use Scale (SUSS) [51], Attitudes Toward Injecting Drug Users (ATIDU) [52], and Health Professionals Attitudes Toward the Homeless Inventory (HPATHI) [53]. Physicians were also asked to provide their contact information so that they could be contacted about follow up data collection and upcoming KT activities.

**Table 3:** Self-reported participant variables (Physicians)

Demographics	• Age
	• Gender
	• Graduation year
	• Discipline (family medicine, emergency medicine)
	• Practice status (resident, practicing physician)
	
Practice behaviour	• Comfort managing patients from target population
	• Perceived success finding common ground with patients from target population
	• Frequency of use of addiction screening
	• Frequency of use of brief intervention
	• Frequency of referral to addiction service providers
	• Frequency of addiction follow-up with patients having completed treatment
	
Attitude	• Short Understanding of Substance use Scale
	• Attitudes Toward Injecting Drug Users scale
	• Health Professionals Attitudes Toward the Homeless Inventory
	
Project feedback	• Presentation use/usefulness
	• Video use/usefulness
	• Electronic module use/usefulness
	• Champions use/usefulness
	• Inner city tour use/usefulness
	• Point of care reminder use/usefulness

The PC contacted physicians who completed the baseline survey at six and twelve month time points to make arrangements for completion of each of the follow up surveys. Identical to the baseline survey, the six month survey was administered to account for any possible interim change in the study group after the patient KT intervention but prior to the physician KT intervention. The 12 month survey was identical to the baseline survey with the addition of items assessing whether or not participants used each KT resource and to what extent each resource was helpful if used.

### Analysis

Baseline and follow-up characteristics of the patient and physician samples were reviewed descriptively. Next, chi squared testing was performed for all post-intervention outcomes of interest by intervention status. Mixed design ANOVA analyses were performed to adjust for potential differences in baseline characteristics and to examine change across time. Additional sub-analyses separating participants in Family Medicine and Emergency Medicine settings were performed.

## Discussion

It is well recognized that vulnerable populations face a “triple threat” – they are more likely to get ill, more likely to have difficulty accessing care, and when they do receive treatment, they are more likely to receive suboptimal care [54]. Despite the huge need for effective interventions in this population, patients with unstable substance use and/or without permanent housing are typically excluded from traditional research studies due to challenges in obtaining informed consent and finding patients for follow up. We would argue that more research and expertise around how to take research findings and translate them into effective interventions for the patients with the highest burden of disease is urgently needed.

Research with vulnerable and marginalized groups, however, requires a specific skill set. Research must be considered culturally safe by the community and must reflect the voice and needs of the participant group. Researchers must believe in the fundamental right of those who are considered vulnerable to be heard and treated with dignity and respect. Research must also be cognizant of the Four P’s: the need for true partnership; participation of the community (including building research capacity); protection from exploitation and the reinforcement of negative stereotypes; and sharing the power within the relationship [55]. Participant studies have shown that mistrust and misconceptions around research are prevalent in drug addiction research and that specific care and attention must be given to building relationships [56,57].

Community engagement in KT efforts may influence their impact significantly, and its importance cannot be understated. Our team has attempted to adhere as closely as possible to CBPR principles, and as a result our protocol has some unique strengths. Our community partnerships have been developed and nurtured over several years of collaboration. We also had direct community input into the project from its inception both through formal and informal partnerships, as well as the hiring of a CC who helped to guide research questions and interpretation. All our team members also spent several weeks of orientation in the community in order to become familiar with community resources, community agencies and community members. Close attention to community partnerships, team membership, and team training has provided tangible benefits to both researchers (e.g. facilitated recruitment and retention) and the community (e.g. research relevance and capacity-building).

Given the nature of KT and the setting for implementation, the protocol also has inherent limitations. Traditional randomization was not possible, and contamination of intervention and control groups could not be fully prevented. Co-intervention from the rollout of physician KT activities was possible for those patient participants recruited later into the study or more difficult to find for follow-up sessions. Physician outcome data relied solely on self-report because of a lack of validated tools available to assess SBIRT fidelity in practice, and because of ethical limitations on the ability to track physician-specific administrative health data concomitantly with patient-specific data.

Rewards and challenges unique to conducting and evaluating KT in a marginalized setting need to be documented. We anticipate that not all project learnings will be quantitative in nature, and that some quantitative findings will benefit from joint interpretation by the community. Qualitative assessments of the project’s impact on the community are underway to ensure that this documentation reflects both the academic and community experience.

The available SBIRT and patient engagement literature suggests that it is time to extend efforts beyond evidence generation and into research on best practices in evidence uptake. This is particularly the case in marginalized health care settings where community collaborations may be occurring but implementation research findings are minimally documented and best practices are therefore hard to reproduce. This CBPR project will contribute to the scientific community’s limited understanding of effective KT in this unique setting.

## Abbreviations

SBIRT: Screening, brief intervention and referral for treatment; SEP: Socioeconomic position; KT: Knowledge translation; CBPR: Community based participatory research; ED: Emergency department; PCN: Primary care network; AUDIT: Alcohol use disorders identification test; DUDIT: Drug use disorders identification test; CC: Community consultant; RA: Research assistant; PC: Project coordinator; SOCRATES: Stages of change readiness and treatment eagerness scale; HCCQ: Health care climate questionnaire; SUSS: Short understanding of substance use scale; ATIDU: Attitudes toward injecting drug users; HPATHI: Health professionals attitudes toward the homeless inventory

## Competing interests

The authors declare that they have no competing interests.

## Authors’ contributions

GS was the study lead and oversaw all aspects of the protocol KT development. KD was the study co-lead and oversaw all aspects of the protocol and KT development, in particular for the emergency medicine setting. CV was the project coordinator and was responsible for protocol implementation, data management, and analysis. SK was the project research assistant and was responsible for recruitment and data collection. KM co-facilitated the development of the patient booklet. TB co-facilitated the development of the patient booklet. MT was a key community collaborator and facilitated community involvement in patient KT development and implementation. RM was a co-investigator and provided guidance on administrative health data collection and ongoing analysis. GC was a co-investigator and provided guidance on knowledge synthesis and KT best practices as well as the protocol itself. TCW was a co-investigator and provided guidance on all aspects of the protocol and KT development. All authors read and approved the final manuscript.

## Authors’ information

GS is an Assistant Professor in the University of Alberta Department of Family Medicine and assistant Director of the Edmonton Inner City Health Research and Education Network.

KD is an Associate Clinical Professor in the University of Alberta Department of Emergency Medicine and co-Director of the Edmonton Inner City Health Research and Education Network.

CV is a project coordinator with the University of Alberta Department of Emergency Medicine.

SK is a project research assistant with the University of Alberta Department of Emergency Medicine.

KM is a registered nurse and a masters student in nursing (Global Health) at the University of Toronto.

TB is an emergency medicine resident at the University of Alberta.

MT is a registered nurse and the program manager for the Streetworks harm reduction program.

RM is a Research and Evaluation Consultant for Edmonton Southside and Edmonton West Primary Care Networks.

GC is a Professor in the University of Alberta Faculty of Nursing and holds a Population health Investigator award from the Alberta Heritage Foundation for Medical Research.

TCW is Professor and Associate Dean (Research) in the University of Alberta School of Public Health. He has received salary support from Alberta Innovates-Health Solutions.

## Pre-publication history

The pre-publication history for this paper can be accessed here:

http://www.biomedcentral.com/1472-6963/13/108/prepub
